# Potential extinction cascades in a desert ecosystem: Linking food web interactions to community viability

**DOI:** 10.1002/ece3.10930

**Published:** 2024-02-15

**Authors:** Adam J. Eichenwald, Nina H. Fefferman, J. Michael Reed

**Affiliations:** ^1^ Department of Biology Tufts University Medford Massachusetts USA; ^2^ Department of Ecology and Evolutionary Biology University of Tennessee Knoxville Tennessee USA

**Keywords:** biodiversity, climate change, community viability analysis, CVA, ecological networks, Mojave Desert, secondary extinction, trophic cascade

## Abstract

Desert communities are threatened with species loss due to climate change, and their resistance to such losses is unknown. We constructed a food web of the Mojave Desert terrestrial community (300 nodes, 4080 edges) to empirically examine the potential cascading effects of bird extinctions on this desert network, compared to losses of mammals and lizards. We focused on birds because they are already disappearing from the Mojave, and their relative thermal vulnerabilities are known. We quantified bottom‐up secondary extinctions and evaluated the relative resistance of the community to losses of each vertebrate group. The impact of random bird species loss was relatively low compared to the consequences of mammal (causing the greatest number of cascading losses) or reptile loss, and birds were relatively less likely to be in trophic positions that could drive top‐down effects in apparent competition and tri‐tropic cascade motifs. An avian extinction cascade with year‐long resident birds caused more secondary extinctions than the cascade involving all bird species for randomized ordered extinctions. Notably, we also found that relatively high interconnectivity among avian species has formed a subweb, enhancing network resistance to bird losses.

## Graphical abstract

This study explores the potential cascading effects of bird extinctions on the Mojave Desert terrestrial community compared to losses of mammals and lizards. Bird species, which are already disappearing from the area, showed relatively low impacts on the modeled network compared to mammal or reptile loss. Avian extinction cascades with year‐long resident birds resulted in more secondary extinctions than cascades involving all bird species, with the network's high interconnectivity among avian species enhancing comparative resistance to bird losses.

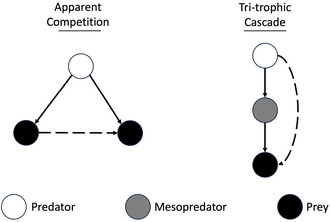

## 1 INTRODUCTION

Investigating the interactions between species can be a key aspect of safeguarding biodiversity and promoting effective conservation efforts (Eichenwald & Reed, 2021; Sabo, 2008; Soulé et al., 2005). The functional loss of even a single species can have far‐reaching and sometimes unforeseen consequences for entire ecosystems (Paine, 1974; Terborgh et al., 2001), in some cases resulting in extinction cascades via secondary extinctions (Brodie et al., 2014; Säterberg et al., 2013). For example, the loss of sea otters (*Enhydra lutris*) famously resulted in the catastrophic collapse of biodiverse kelp forests into urchin barrens (Estes & Palmisano, 1974). The effects of species loss may pose threats to human health (Markandya et al., 2008), or to ecosystem services (Pike & Mitchell, 2013). As such, conservation efforts often must go beyond focusing on individual species and instead incorporate an understanding of their interactions within a community (Soulé et al., 2005; White et al., 2013; Zipkin et al., 2010). Doing so can enable us to better predict the effects of environmental perturbations (e.g., Jönsson & Thor, 2012), mitigate the spread of introduced, invasive species (Galiana et al., 2014), promote ecosystem services (Buechley & Şekercioğlu, 2016), and rehabilitate degraded landscapes (Soulé et al., 2003). Considering the intricate and indirect relationships between species becomes even more crucial in the face of ongoing challenges such as climate change, which result in negative effects compounding unpredictably across complex systems (Tylianakis et al., 2008). Neglecting to consider the sometimes intricate and indirect relationships between species can lead to management approaches that are ineffective or even detrimental to an ecosystem (Bowen & Lidgard, 2013; Johst et al., 2006; Letnic & Koch, 2010; McDonald‐Madden et al., 2016).

One promising approach to addressing these challenges is through community viability analysis (CVA) (Ebenman et al., 2004; Ebenman & Jonsson, 2005; Eichenwald & Reed, 2021). CVA encompasses a variety of approaches to quantifying community structure, composition, and function in response to perturbations or management actions. For instance, by representing a community as a network of species interactions, researchers can identify species that play a significant role in community stability or resilience (Eichenwald & Reed, 2021; Jönsson & Thor, 2012), or identify effective management interventions (McDonald‐Madden et al., 2016).

Here we conduct a resistance‐based community viability analysis (Eichenwald & Reed, 2021) of the terrestrial community of the Mojave Desert, a well‐studied ecosystem in the southwest United States (e.g., Iknayan & Beissinger, 2018; Kissel et al., 2023; Rundel & Gibson, 1996a, 1996b, 1996c, 1996d, 1996e). We did this by first constructing a food web and then examining the potential cascading loss of species following initial species losses. Specifically, we assess the impact of bird loss compared to losses of mammals and lizards. which despite known susceptibility to climate change (e.g., Sinervo et al., 2010) have been found to be more resistant in the Mojave Desert in comparison to birds (Riddell et al., 2021). The Mojave Desert bird community has suffered notable declines over the last century that have been attributed to climate change (Iknayan & Beissinger, 2018; Riddell et al., 2019, 2021). In contrast, mammal species in the Mojave have remained relatively stable despite increasing heat, presumably because they dig burrows, which function as thermal refugia (Riddell et al., 2021). Despite a century of research in this ecosystem, there has been limited investigation into the extent to which the loss of different species may affect the persistence of others.

To investigate the vulnerability of the Mojave Desert community to bird loss compared to losses of other vertebrates, we constructed a food web for the terrestrial community and conducted network analyses. Network analyses can be used to map and model interspecific relationships (Borrett et al., 2014) such as food webs, where links between species indicate one consuming the other (de Visser et al., 2011; McDonald‐Madden et al., 2016). We quantified bottom‐up secondary extinctions to capture potential cascading effects of the increasing frequency of primary extinctions of birds, mammals, and reptiles on other species in the food web (Dunne & Williams, 2009). Because birds are declining in the Mojave, we analyzed the effects of bird loss in order of thermal vulnerability compared to random loss of species. These analyses provided insights into the relative importance of each vertebrate group in maintaining community structure and stability in the face of species loss. Additionally, we investigated how vertebrate groups contribute to food web patterns linked to top‐down secondary extinctions (Baiser et al., 2016). These patterns, referred to as motifs or subgraphs, were assessed for their frequency of occurrence among mammals, reptiles, and birds. This analysis provides insights into the relative importance of these vertebrates in trophic interactions, providing further understanding of their potential impacts on the community.

## 2 METHODS

The Mojave Desert spans broad latitudinal (34.8° to 36.2°), longitudinal (−117.2° to −115.8°), and elevational ranges (−82 to 3367 m), and tends to share much of its biota with the neighboring Sonoran Desert to the south and the Great Basin to the north (Rundel & Gibson, 1996a, 1996b, 1996c, 1996d, 1996e). Daytime temperatures are generally warm, and the desert holds the record for the highest measured air temperature on the planet (El Fadli et al., 2013). However, the Mojave may experience cool Arctic air masses during the winter rainy season and hence receives some snow (Rundel & Gibson, 1996a, 1996b, 1996c, 1996d, 1996e). The desert has experienced a rise in mean annual air temperature by approximately 2°C since the early 20th century (Bai et al., 2011). We developed our particular CVA of the Mojave Desert biotic community following the overarching guidelines proposed by Eichenwald and Reed (2021): (1) delineate the focal community, (2) decide on viability measures and questions, (3) enact calculations, and (4) address uncertainty.

### 2.1 Delineating the focal community

Food webs have been generated for some communities in the Mojave Desert, such as soil nematodes (Ferris, 2010) and aquatic systems (Wilson & Blinn, 2007). However, we needed to create a food web containing the terrestrial vertebrate community of the Mojave Desert for our analyses. The first step was to assemble the most complete list of taxa available for the study region. Historically, such a task was performed by consulting with local experts (Martinez, 1991), an approach that could result in incomplete webs with missing species (Polis, 1991). More recently, online databases collated from large‐scale citizen science observations can allow construction of more thorough taxa lists, although such catalogs still tend to be deficient when it comes to insect and plant species. We constructed our taxa list by downloading observation data from the Global Biodiversity Information Facility (GBIF, gbif.org, April 2023) within the geographical limits of the Mojave Desert. We limited data to observations only; although GBIF includes museum specimens in its database, the latitude and longitude associated with these records sometimes are of the museum that currently holds the specimen instead of where the specimen was collected. We then culled the taxa list further by examining the resulting species list one at a time and removing species if they did not actually appear in the Mojave Desert, based on species‐specific distributional accounts from the US Geological Survey (USGS) Gap Analysis Program (GAP) (McKerrow, 2018). There is a variety of reasons for these species being incorrectly included in the Mojave Desert in the GBIF database, such as mapping or identification errors (Roberts et al., 2010; Zizka et al., 2020). For example, the white‐tailed antelope ground squirrel (*Ammospermophilus leucurus*) looks very similar to the San Joaquin antelope squirrel (*A. nelsoni*); however, the latter has a restricted range that does not include the Mojave Desert, while the former is ubiquitous in the Mojave. Consequently, when the San Joaquin antelope squirrel appeared in our first‐pass taxa list, we culled it on the assumption that it was a misidentification or misapplied location. We did not have to rely on this method to obtain a list of bird species, as we had already created this list in a previous paper (Eichenwald & Reed, 2023) (although we did remove the hairy woodpecker *Leuconotopicus villosus*, though its range covers some of the Mojave, it is so scarce within that range that we could not justify including it in the web). We classified birds into two categories for the extinction cascade (see below): non‐residents and residents. We classify resident birds are present year‐round in the Mojave Desert, while we classify non‐resident birds are present only during the pre‐breeding (migration) or breeding season, as classified by the Cornell Lab of Ornithology's ebirdst models (Strimas‐Mackey et al., 2021). We focused on birds that are present during the summer, as heat exposure from climate change is thought to be the major driver of Mojave avian species loss (Riddell et al., 2021). Therefore, birds that are present only during the winter or in the post‐breeding season are not included in our web.

Assigning predation links between species is more time‐consuming. In recent years, some researchers have attempted to derive feeding links from patterns of co‐occurrence and trophic levels; however, co‐occurring species do not necessarily interact with one another in expected ways (Blanchet et al., 2020). Alternative methods of inferring feeding links based on predator and prey body mass and trophic level have varying degrees of success (Freilich et al., 2020) but can still result in inaccuracies (Rohr et al., 2017). To minimize errors of inclusion in food‐web links, we chose to include only feeding links that have been confirmed (Martinez, 1991) instead of inferring feeding links. For each vertebrate species, we searched the literature, including compendium volumes such as Birds of the World (Billerman et al., 2022) for evidence (e.g., observations of feeding, investigation of stomach contents, eDNA) of its predators and prey. Each species' common and scientific names were used as keywords in Google Scholar, along with the keywords “stomach contents,” “food,” “diet,” “predation,” “consumption,” or “prey.” If a species' scientific name was changed in the last few decades, we performed separate searches with older names as keywords as well. We also searched for science‐based encyclopedias or compendia on specific taxa (e.g., “reptiles”) in the Americas. Finally, we consulted the Global Biotic Interactions database (Poelen et al., 2014) and the Avian Diet Database (Hurlbert et al., 2021), which are archives of consumption links. Observations from these interaction‐specific data sources were supplemental; we did not assume that all feeding interactions present in the Mojave Desert were available in such sources.

We did not include generic links, such as if a species is said to consume “mammals,” we did not include all mammals in that species' diet. However, if a predator is confirmed to eat animals from a particular genus, we did include links to all species within that genus. Some feeding links were observed but not included in the final network because they were likely atypical events. For instance, a black‐chinned hummingbird (*Archilochus alexandri*) was once observed hovering in front of the nose of a captive mountain lion (*Puma concolor*) and the cat consumed the bird (Baltosser & Russell, 2020). We considered this unlikely to be relevant in a natural food web. We also scrutinized links attributed via DNA analyses of gut contents or feces. When a predator eats another animal, it is possible for the DNA of anything the prey consumed to also appear in the predator's stomach (Sheppard et al., 2005). Alternatively, some herbivores scavenge meat from carcasses or engage in occasional predation (Pietz & Granfors, 2000), which can result in particularly strange DNA‐verified feeding links. Therefore, if a feeding link appeared unlikely and was verified by DNA, we did not include it in the final web. For example, a known omnivore such as the coyote (*Canis latrans*) consuming plant matter is strange but not unheard of, and so such links were included in the web. Chukar (*Alectoris chukar*), on the other hand, subsist on leaves and seeds and feed their young insects; therefore, predation links suggesting chukar hunt rodents (e.g., Hurlbert et al., 2021) were not included. Although we were able to find data on predators and prey for each vertebrate species, similarly detailed information for insects and plants was less available. We found that data on feeding interactions involving these groups was generally provided at higher taxonomic levels (e.g., order, class), making it impossible to include them in a food web at the species level. Insects and arachnids, therefore, were aggregated at taxonomic orders; this type of aggregation is common in published food webs (Martinez, 1991). Plants were aggregated at taxonomic orders for all calculations that did not require interaction strength (see below for explanation on interaction strength) but were aggregated at the taxonomic kingdom level for calculations involving biomass (see below).

Thus, we generated a food web where directed links represent a known consumptive interaction between predator and prey. However, not all interaction links are equivalent; in fact, differences in interaction strength across a web are common based on the percent of a species' diet coming from each prey type (Berlow et al., 2004). Even poorly estimated interaction strengths provide greater average certainty in modeled predictions based on the web than does an approach that uses only the presence or absence of each interaction (Novak et al., 2011). Therefore, we estimated interaction strength for all links using the fluxweb package in R, which allows for the calculation of energy fluxes in food webs based on the conceptual framework of the “food web energetics” approach (Gauzens et al., 2019). To calculate energy fluxes between predator and prey, the package's functions require three variables: (1) species biomasses in mass per unit area, (2) metabolic rates, and (3) feeding efficiencies. Metabolic rates and feeding efficiencies are calculatable from body mass and species type (vertebrate endotherm, vertebrate ectotherm, invertebrate, plant) of the species in question (plant metabolic rates are considered non‐existent) using equations in the fluxweb package (Gauzens et al., 2019). Estimations of species biomass (g/km^2^) are more difficult, requiring estimations of both individual body mass and number per unit area. Calculating the average mass of each species is straightforward, as the masses of museum specimens are often measured before they are taxidermied. We obtained average vertebrate masses from rvertnet (Chamberlain, 2021). It is also possible to obtain an estimation of species densities (number of individuals per km^2^) allometrically, as Damuth (1981) reported a size‐density relationship (SDR) following a power law with a scaling exponent close to −0.75 (Isaac et al., 2013). This relationship is less accurate at the local scale (White et al., 2007), particularly since the densities of desert species vary greatly due to episodic pulses of productivity. However, it is still a suitable way of approximating species density in comparison to the time‐consuming method of surveying the abundance of each species individually, so long as we recognize that our results are approximate. We allometrically calculated the densities of all vertebrates and multiplied by their masses to obtain species‐by‐species biomass.

Calculating insect and plant biomasses are more difficult due to the aggregation of their nodes, wide variation in masses (for invertebrates), lack of research on allometric relationships, and unclear total numbers of species. Fortunately, there has been a prior survey of arthropod biomasses in some parts of the Mojave Desert, where the results were aggregated by taxonomic order (Rundel & Gibson, 1996a, 1996b, 1996c, 1996d, 1996e). We collected biomass information from this source; however, not all arthropod taxa in our food web had corresponding biomasses in the surveys. Therefore, we used the Rphylopars package in R (Goolsby et al., 2017) to infer missing biomass data. Rphylopars uses statistical models to predict what a missing trait of a species might be based on information about related species, analyzing the evolutionary relationships between species and the traits they possess. The package considers the phylogenetic relationships between species, which helps to account for the fact that closely related species are likely to have relatively similar traits. We constructed a phylogenetic tree using taxize (Chamberlain & Szöcs, 2013) to represent the evolutionary relationships among species of interest, then used this tree as input data for the Rphylopars package. We employed a Brownian motion evolutionary model to estimate missing trait values for species lacking data, which is the default and most‐used method (Goolsby et al., 2017). Our method of calculating network extinctions (next section) was insensitive to differences in insect biomass within their confidence intervals (see next section). Therefore, we assumed that each arthropod node had the median biomasses calculated by Rphylopars.

We were unable to find reports that provided above‐ground plant biomass in the Mojave Desert, even at a high taxonomic level. However, Rundel and Gibson (1996a, 1996b, 1996c, 1996d, 1996e) provided an equation relating average total aboveground biomass in g/m^2^ to the amount of precipitation from September through March in the Mojave Desert:

$$\begin{array}{c} \log\left(\text{Aboveground}\;\mathrm{Net}\;\text{Production}\right)=\\ \;1.976\;\log\left(\text{SepttoMarchPrecip}-26.2\right)-2.746 \end{array}$$

We used this equation, including total September–March precipitation across the years of their study (1964–1968, 1971–1976), to estimate above‐ground plant biomass.

### 2.2 Viability measures, calculations

To investigate the potential for secondary extinctions of vertebrates (where the loss of one species leads to the losses of others), we utilized the NetworkExtinctions package in R (Ávila‐Thieme et al., 2023), which is based on methods originally proposed by Dunne and Williams (2009). The method involves removal of a primary species from the network, followed by a review of the resources remaining for the remaining species. If any of the remaining species lose all their resource species, they are removed from the web, resulting in a secondary extinction. However, species can be forced into extinction after losing even a few of their resources, depending on how reliant the consumer was on said resource (Berg et al., 2015). To incorporate the effect of interaction strength in the extinction cascade, we ran the simulation four times for each removal scenario, each with a different threshold value of 0.6, 0.7, 0.8, and 0.9, respectively. This means that a species needed to have a remaining interaction strength between it and all its prey greater than or equal to the threshold value to avoid secondary extinction. For example, a threshold value of 1.0 would cause a predator to go extinct if it lost a single prey species (plants are included as prey), while a threshold of 0.0 would result in a predator never going extinct even after losing all its prey.

The predicted extinction orders for Mojave mammals, lizards, and birds based on increasing temperatures have not been evaluated. Thus, we randomly sorted the order of mammal removal from the food web, computed the resulting secondary extinctions, and repeated this process 100 times to account for variation in the order of removal. We tested the effect of reptile and bird removal from the community using the same randomization procedure. For birds, we repeated this procedure three times. The first removal experiment included all bird species. In addition, because we suspected that losing year‐round resident bird species would have a different effect on the system than would losing birds that lived in the Mojave only part of the year, we conducted two additional removal experiments: removing only non‐resident bird species and removing only resident bird species. We did not include plants and invertebrates in a primary removal cascade due to their aggregation, which would lead to an artificially high change in the number of links and render any comparison incomplete or invalid. We also evaluated the degree to which predicted extinction cascades were sensitive to inferred arthropod biomasses from Rphylopars. For each arthropod with inferred biomasses, we randomly selected biomasses from within the 95% confidence interval calculated by Rphylopars to parameterize the interaction strengths for the food web and then ran the predetermined climate change‐induced primary extinction cascade with all birds. We ran this simulation 100 times with the same bird extinction order, but different arthropod biomass values drawn randomly from their distribution. The resulting secondary extinction cascade for each run was always the same. This was unsurprising, as the extinction cascade method is based on bottom‐up effects, and arthropods are on the lower end of the food chain and were not included in primary removal cascades. As randomly choosing biomasses from within the confidence interval did not influence our cascades of interest, we used the median inferred biomass for arthropods as listed above.

To gain a deeper understanding of potential bottom‐up secondary extinction cascades within our food web, we examined homophily between nodes within and between different ecological groups (mammals, plants, insects, birds, and reptiles). Homophily helps us see whether species with similar characteristics tend to connect and interact more often than those that are different (e.g., are mammals more likely to interact just with other mammals, or do they link to other animal types as well?). We employed Coleman's Homophily Index (Coleman, 1958), which calculates homophily scores within each defined group. The index gives us a number between −1 and 1: a score of 0 means there are equal connections between different groups, 1 means all connections are within the same group, and −1 means all connections are between different groups. As a reminder, our secondary extinction cascades operated from prey to predator, whereas the homophily index traditionally measures connections from predator to prey. Therefore, a homophily index would provide information on a top‐down cascade. To obtain information on bottom‐up effects, we inverted the links in the graph before calculating the homophily index.

**FIGURE 1:**
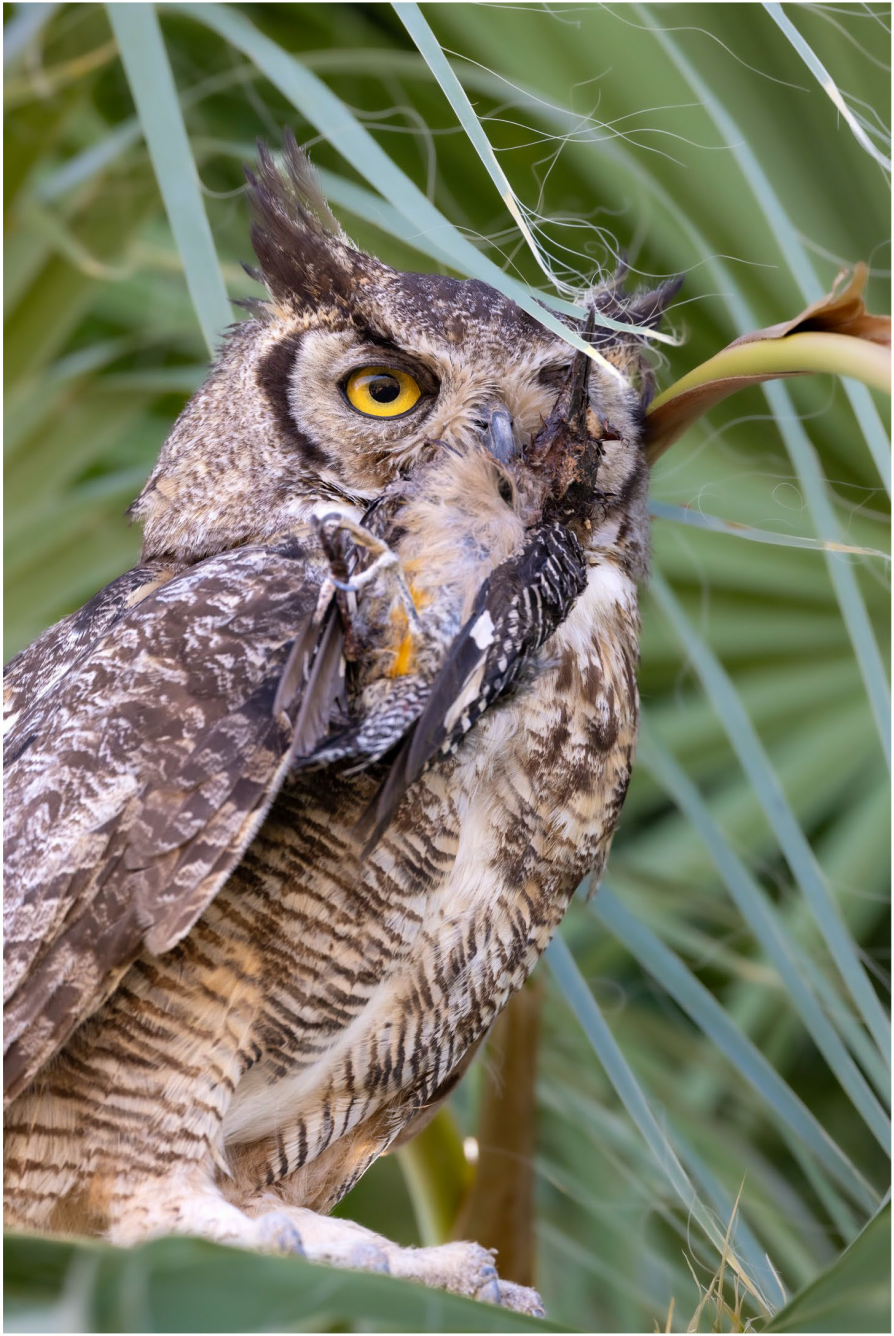
A Great Horned Owl (*Bubo virginianus*) depredates a woodpecker (*Picidae*) in the desert. Photo taken by David Krauss.

**FIGURE 2:**
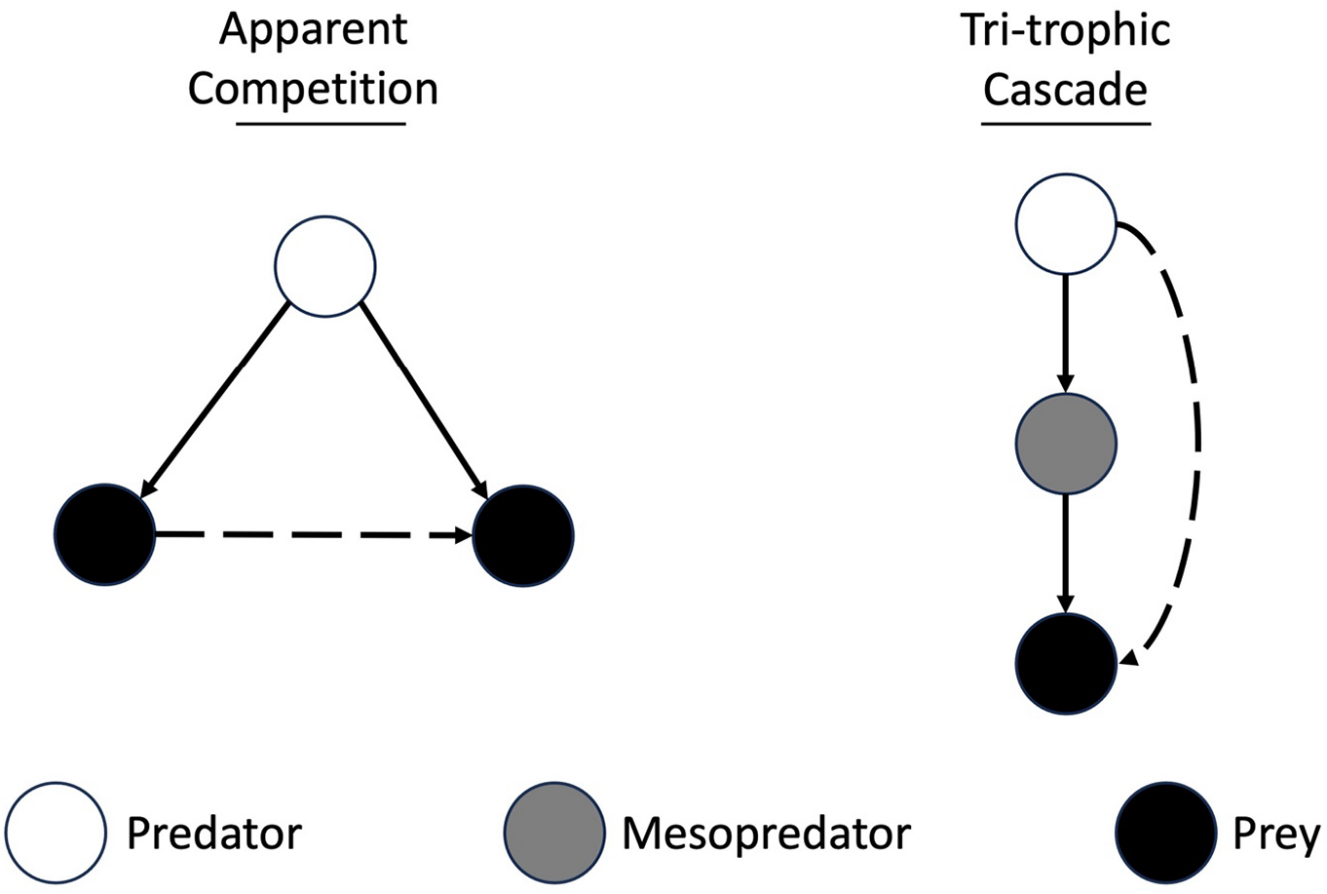
Example networks demonstrating the shape of apparent competition and tri‐trophic cascade subgraphs. Predator/prey (consumptive) interactions are represented by solid lines, while indirect effects are represented by dashed lines.

While the NetworkExtinctions approach is valuable for analyzing food webs, it has limitations in accurately predicting the impact of consumer loss on the resources that these consumers utilized. This is because it can capture only extinctions caused by bottom‐up effects, such as the primary loss of all or a fraction of a consumer's resources. It does not consider the potential for top‐down effects, such as the impact of predators (Berg et al., 2015; Terborgh et al., 2001). Consequently, methods investigating secondary extinctions in topological food webs may overestimate the network's robustness by overlooking the influence of predators (Curtsdotter et al., 2011). However, within food webs, patterns of interactions referred to as motifs or subgraphs (used synonymously here) are observed (McLeod & Leroux, 2021). These motifs vary in their characteristics, and some specific subgraphs are facilitative of top‐down effects. For instance, apparent competition occurs when two species that do not directly compete for resources affect each other indirectly by acting as prey for the same predator. This interaction forms a triangular motif (Holt & Bonsall, 2017; Figure 1). In the event of the extinction of one prey species, the predator may compensate by increasing its consumption of an alternative prey, potentially leading to a secondary extinction. It is worth noting that in some cases, apparent competition might not be strong enough to result in a secondary extinction, particularly if the extinct prey species is a minor or relatively opportunistic component of the predator's diet or if there are many species in the predator's diet. Nevertheless, within our food web (assuming completeness), the set of all apparent competition‐shaped subgraphs *A* encompasses the number of apparent competition motifs that may lead to secondary extinctions *B* (*B* is a proper subset of *A*, or *B*⊊*A*). By assuming a proportional relationship between A and B, we can assess the relative importance of a taxon based on the frequency of its species appearing in trophically key positions within subgraphs (McLeod & Leroux, 2021). The same logic applies to trophic cascades, where predators can exert indirect effects on species through the control of intermediate consumers (Ripple et al., 2016). Trophic cascades are also characterized in their shape by easily recognizable subgraphs (Ebenman & Jonsson, 2005; Figure 2). If an apex predator is removed from the web, the intermediate consumer that it used to control may experience population growth and cause severe depletion of its own prey species.

We identified all apparent competition and trophic cascade subgraphs in our food web using the VF2 algorithm (Cordella et al., 2004) in the R iGraph package (Csardi & Nepusz, 2006) (this method is also known as subgraph enumeration). We limited trophic cascade subgraphs to tri‐trophic food chains, excluding chains where the apex consumer depredated both the intermediate consumer and the prey of the intermediate consumer. We tallied the frequency of subgraphs where birds, mammals, and reptiles were identified as either the apex predator (for trophic cascade motifs) or the prey (for apparent competition motifs). This allowed us to quantify the occurrences of these motifs specifically associated with each taxonomic class. We then compared the occurrences of these motifs per class to Erdős–Rényi algorithm‐generated networks, which we used as a null model (sensu Baiser et al., 2016). This algorithm generates random networks where the only constraint is that the randomized network must have the same number of nodes and expected links as the observed network (Erdös & Rényi, 1959). We ran the null model 100 times, counting and splitting subgraphs in the same way as with the empirical data. We then compared empirical motif counts to those of the null models using z‐scores and p‐values (Baiser et al., 2016). We focused on motifs where an animal's removal from the web could result in a top‐down secondary extinction. For apparent competition‐based subgraphs, for example, the prey positions should be the cause of top‐down forced secondary extinctions, due to compensatory predation following the loss of one of the species. For tri‐trophic cascade‐based subgraphs, the apex predator position should be the cause of top‐down forced secondary extinctions from the release of the second‐tier consumer from predatory control. We refer to these positions in the motifs as driving positions (or drivers).

## 3 RESULTS

**FIGURE 3:**
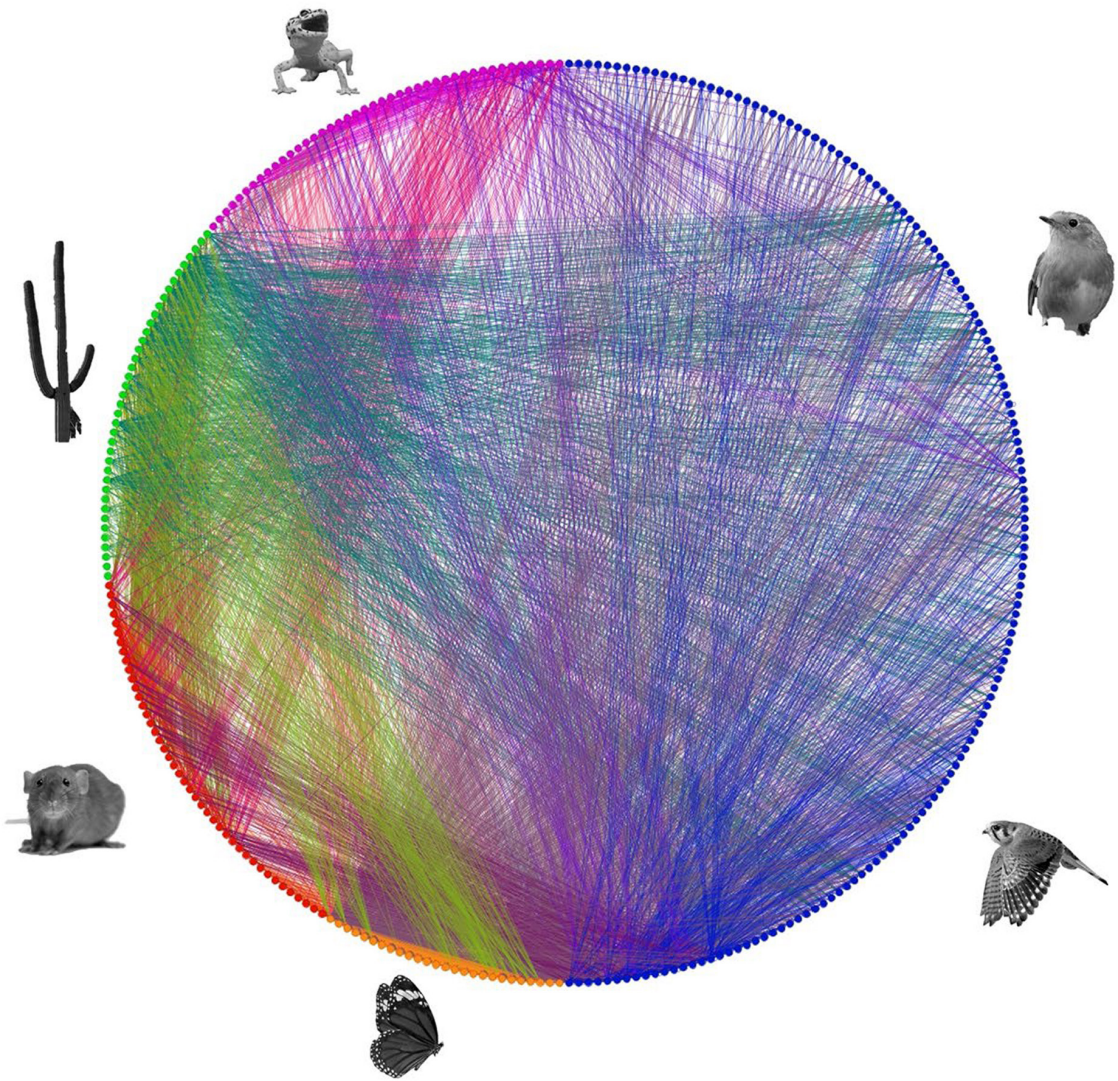
The full food web of the Mojave Desert terrestrial community created and used in this study. Plants are shown in green, mammals in red, insects in orange, birds in blue, and reptiles in purple. The color of the line matches that of what is being consumed, (e.g., a bird eating a plant will be joined by a green line). This web has 150 birds, 43 mammals, 42 reptiles, 26 insects (aggregated to order), and 39 plants (aggregated to order). There are 4080 feeding links.

Our Mojave Desert food web comprised of 150 bird species, 43 mammals, 42 reptiles, 26 orders of insects, and 39 orders of plants. There were 4080 edges in the web (Figure 3; the adjacency matrix and list of predation links are found in Tables S1 and S2). The mean number of links a node had for birds was 20.7 ± 17.4 (median 16.0), for mammals it was 23.5 ± 13.3 (median 18.0), 23.0 ± 14.3 (median 18.5) for reptiles, 72.4 ± 69.6 (median 42.5) for invertebrates, and 30.6 ± 24.4 (median 26.0) for plants. For trophic levels of 1 to 4, where 1 is plants and 4 is apex predators, birds had an average trophic level of 3.24 ± 0.10, mammals had an average trophic level of 2.85 ± 0.23, reptiles had an average trophic level of 3.75 ± 0.17, and invertebrates had an average trophic level of 2.96 ± 0.32.

Most consumptive interactions with birds involved other birds, invertebrates, and plants (including interactions where birds were either predator or prey). In contrast, mammals and reptiles exhibited more balanced interactions with other animals in the web. Birds demonstrated a relatively high homophily score of 0.66, indicating a strong inclination for birds to form connections with other birds within the food web. In contrast, the invertebrate and mammal groups exhibited lower homophily scores of 0.02 and 0.07, respectively – values indicative of an even mix of links within and without their groups. The reptile group displayed a homophily score of 0.36, indicating a moderate tendency relative to the other groups for reptiles to be connected to other reptiles within the food web. Plants, as primary producers, did not act as predators or prey for other plants in the food web, resulting in a homophily score of −1.00.

**FIGURE 4:**
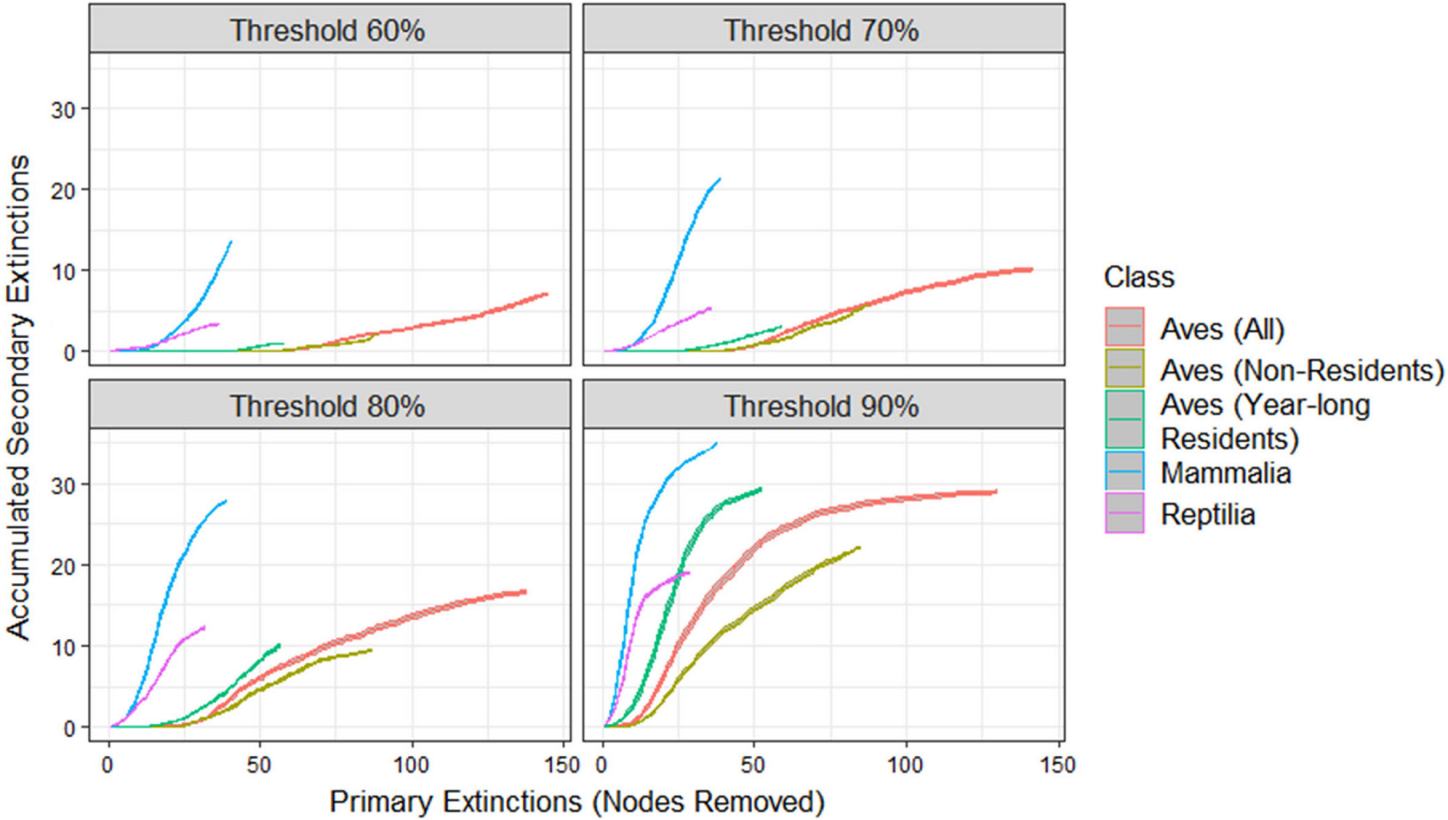
Secondary extinction cascades caused by primary extinctions from birds (all species, resident‐removals only, and non‐resident‐removals only (where non‐residents are birds that either breed or migrate through the Mojave but are not present year‐round)), mammals, and reptiles. All cascade lines represent 95% confidence intervals based on randomized order of species removal from 100 replicates. Threshold percentages means that a species needed to have a remaining interaction strength greater than or equal to the threshold following a primary extinction to avoid secondary extinction (a threshold of 100% would always result in secondary extinctions, while a threshold of 0% never would).

All instances of bird primary extinction cascades resulted in fewer accumulated secondary extinctions than observed from the extinctions of either reptiles or mammals under all threshold (remaining interaction strength) scenarios (Figure 4). In fact, under the 60% and 70% threshold conditions, random loss of bird species did not cause any secondary extinctions until over 50 species were lost from the food web. Mammal extinctions resulted in the most rapidly accumulating number of secondary extinctions, as well as the greatest number of accumulated secondary extinctions in the food web. Extinction of reptiles from the food web resulted in a rate and accumulated number of secondary extinctions that was intermediate between birds and mammals. An avian extinction cascade where only birds that were year‐long residents were removed also resulted in greater numbers of accumulated secondary extinctions than did than the cascade from extinctions that included all bird species (Figure 4). However, cascades including only birds that were in the Mojave for part of the year (non‐residents) resulted in similar levels of secondary extinctions to the cascade that included all birds except under higher thresholds. At thresholds of 80%–90%, randomized order of extinction of bird species resulted in higher numbers of secondary extinctions when all bird species were included compared to the effects of randomized extinctions from only non‐residents (Figure 4).

**FIGURE 5:**
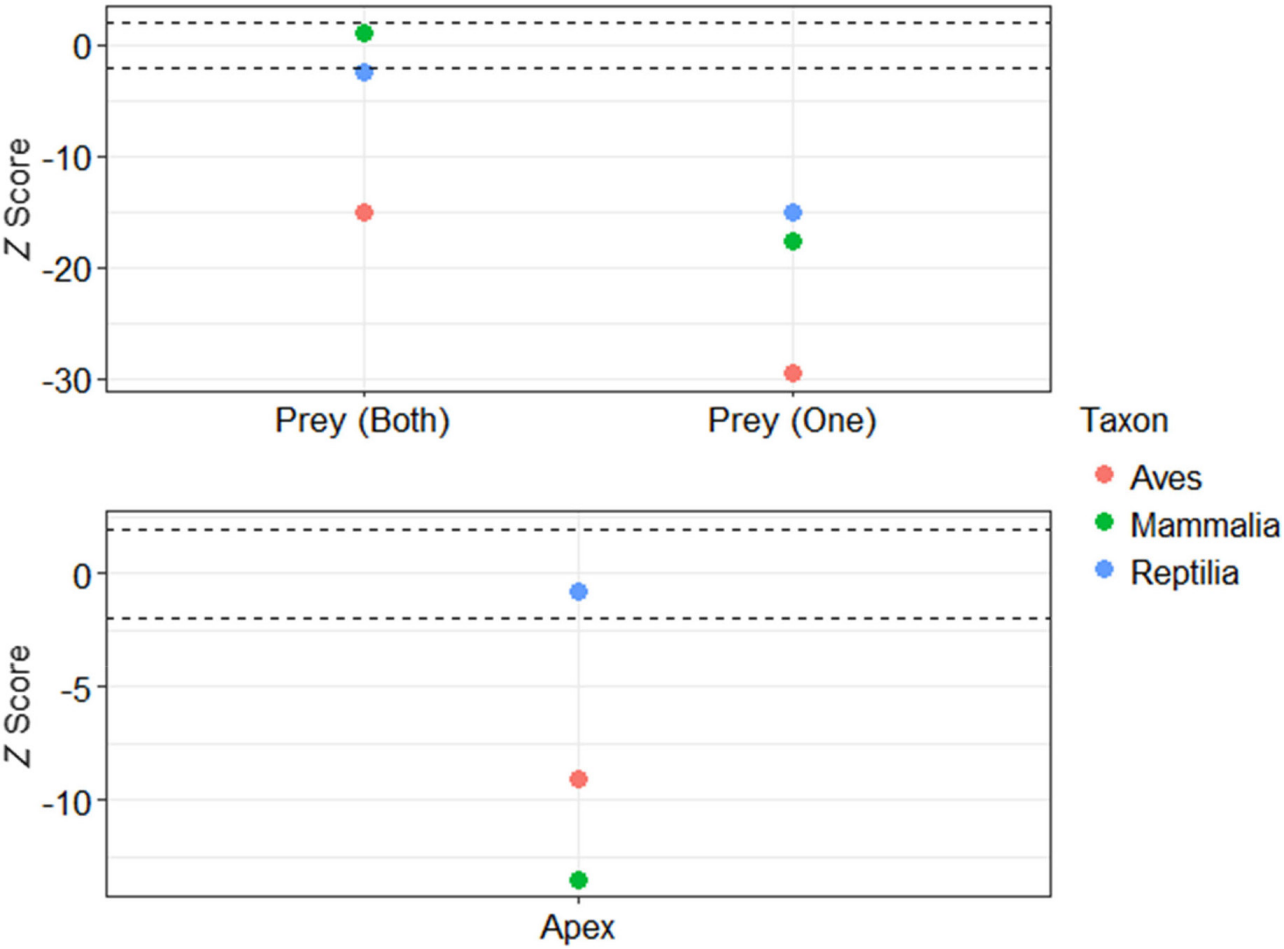
*Z* scores of the number of apparent competition (top) and tri‐trophic (bottom) motifs in comparison to null graphs. *X*‐axis labels refer to the driving position (positions that can be the cause of top‐down forced secondary extinctions) within the motif (for example, the *Aves* point over *prey (both)* in the top graph refers to the *z*‐score where both prey in the apparent competition motif were birds). We only count motifs where the taxon in question is in driving position, where losing that species could result in top‐down driven secondary extinctions. Points between the dashed lines are not significantly different from the null model; points outside the dashed lines are significantly different from the null.

There were significantly fewer birds than expected in driving positions (i.e., positions that can be the cause of top‐down forced secondary extinctions) in apparent competition‐based subgraphs when compared to null models, including motifs where only one of the prey species was a bird (*z* = −32.1, *p* < .001) and those where both prey species were birds (*z* = −15.1, *p* < .001). However, comparing z‐scores reveal that mammals and reptiles were approximately equally likely to appear as only one of the prey species, and were much more likely to do so than birds (Figure 5). In fact, in motifs where reptiles were both prey species there was no significant difference between real‐world data and the null models (*z* = −0.7, *p* = −.23), while mammals had significantly greater representation than we would expect from null models (*z* = 4.74, *p* = .001, Figure 5). Motifs where reptiles were only one of the prey species were also found less frequently than in null models (*z* = −17.3, *p* < .001), as were mammals (*z* = −18.2, *p* < .001, Figure 5).

There were also significantly fewer birds than expected in driving positions (i.e., the apex predator) in tri‐trophic‐based subgraphs when compared to null models (*z* = −9.1, *p* > .001, Figure 5). There were also significantly fewer mammals than we would expect in the apex position (*z* = −13.5, *p* < .001), but there was no difference between null model expectations and the number of reptiles in the apex position (*z* = −0.73, *p* = .23, Figure 5). Birds were more likely to be found in the apex position than were mammals, but they were comparatively less likely to be in the apex position than were reptiles *(*Figure 5).

## 4 DISCUSSION

Ecosystems are intricate networks of interactions in which the presence or absence of species has the potential to trigger a chain reaction of cascading secondary extinctions throughout the community (Ebenman et al., 2004). Modeling these cascades in real‐world food webs can provide valuable insights for adjusting species management or harvesting strategies (Ávila‐Thieme et al., 2021; de Visser et al., 2011). Indeed, the economic advantages of the ecological network‐based predictive approach has led to its adoption by fisheries scientists (Yun et al., 2017), who not only have developed complex, dynamic representations of marine ecosystems (Fulton et al., 2011) but also have created bespoke software to analyze such models (Heymans et al., 2016). However, studies of this nature in natural terrestrial systems, particularly those that investigate secondary extinction cascades, are rarer despite their potential to predict ecosystem robustness to species loss (Ebenman, 2011). Here we constructed the most comprehensive food web available for terrestrial species in the Mojave Desert as of this publication and used it to test scenarios of vertebrate extinction. We found that the impact of bird species loss on the subsequent structure and richness of the food web via secondary extinction cascades was relatively low compared to the potential consequences of removing mammals or lizards.

The homophily indices we calculated offer valuable clues to the underlying reasons behind these findings. A high proportion of bird links in our network are to other birds, while reptiles moderately connected to other reptiles and mammal connections were proportionally equal. The high interconnectivity among avian species has resulted in the formation of a “subweb,” a subset of species that are highly connected to prey and/or predators within the same subset (Melián & Bascompte, 2004). Such groupings can enhance the network's resilience and protect it from the impacts of losing highly connected species from within the subweb (Melián & Bascompte, 2002, 2004), insulating the food web from bird extinctions and demonstrating why other vertebrates have greater import to network persistence in the Mojave Desert.

Evidence from published research corroborates our conclusion. Riddell et al. (2021) found that Mojave mammal populations have remained stable despite crashing bird populations, which is suggestive of limited secondary extinctions in this system following avian declines. Furthermore, although there are many examples of cascading effects stemming from apex avian predators around the world (Terraube & Bretagnolle, 2018), Estrada and Rodriguez‐Estrella (2016) explain that in the Baja California Peninsula desert (neighboring the Mojave) such birds are poor surrogates for other species in the area. In fact, they suggest that there is reduced interaction strength between apex birds and their prey in this system, which we note would also reduce the probability of secondary extinctions following species loss.

We add to their supposition on interaction strengths with data from our motif analysis: in this system, there may be less of an opportunity for birds to influence the food web from the top. Birds occupied driving positions within apparent competition‐based motifs less frequently than mammals or reptiles, suggesting they are less likely to cause secondary extinctions through top‐down apparent competition‐based effects. On the other hand, birds occupied the apex predator position in a tri‐trophic motif more frequently than mammals (but less often than reptiles). Therefore, we assume the extinction of a bird species to have a higher likelihood of causing secondary extinctions through top‐down trophic cascade‐based effects compared to mammals, but lower than if a reptile were to go extinct. This result is because mammals in the web are less likely to appear at the top of the food chain and initiate top‐down control of tri‐trophic motifs. Indeed, mammals in our food web are on average 0.5–1.0 trophic levels lower than the other vertebrates.

The relatively lower importance of birds in the desert ecosystem may be a characteristic of desert life. Deserts typically support few resident bird populations, with only 3% of the total avian breeding population in the central 70% of Australia being characteristic of the region (Keast, 1959; Rundel & Gibson, 1996a, 1996b, 1996c, 1996d, 1996e). Although the Mojave Desert hosts many transient and seasonal bird species, there are only 31 avian species classified as true desert residents across all North America (MacMahon, 1979). During periods of drought in Mojave Desert scrub, the number of resident bird species is notably low (Rundel & Gibson, 1996a, 1996b, 1996c, 1996d, 1996e). We speculate that species in the Mojave Desert relying on the presence of birds for increased survival may be outcompeted by those adapted to thrive in their absence, leading to the avian subweb observed in our results. The limited residency of birds in deserts may also explain why a meta‐analysis did not find evidence of cascading effects from birds in tri‐trophic food chains in desert biomes, as reported by Mäntylä et al. (2011).

Indeed, the relative resilience of our network to bird extinctions does not appear to be replicated in other studies outside of deserts. For example, although Brazilian forest webs are robust to random bird extinctions, the avian species at higher extinction risk are critical in maintaining community structure (Vidal et al., 2014). Indeed, the vulnerability of certain species is often linked to their functional roles and interactions within the network, and random extinctions are less likely to disrupt critical links than when a vulnerable species is lost (Berg et al., 2015). This differs from our findings, where losses of birds at high risk of extinction from climate change had limited to no subsequent effects on network composition or structure. Alternatively, this difference may be attributable to birds having a greater effect on plants and insects through top‐down predation (Mäntylä et al., 2011; Vidal et al., 2014). Since we had to aggregate plants and insects due to limited data availability and were restricted in our capacity to analyze top‐down effects, it is possible that we underestimated the capacity of birds to influence this food web. Furthermore, although we focus on predation, there are other interaction types present in the Mojave that could influence secondary extinction cascades. Gopher tortoises (*Gopherus* spp.), for example, are considered as keystone species due to their propensity to dig burrows that other animals could then use as refugia (Catano & Stout, 2015), and populations of the Mojave Desert tortoise (*G. agassizii*) have declined dramatically over the course of the late 20th and 21st centuries (Kissel et al., 2023).

We caution that a community viability analysis based on food webs without dynamics can underestimate the risk and number of secondary extinctions (Ebenman & Jonsson, 2005). Indeed, topological analysis always predicts a lower number of secondary extinctions than dynamic analysis, especially for food webs with high connectance (Eklof & Ebenman, 2006), while non‐standard food sources may be more common during circumstances that would otherwise result in cascading community failure. Our web lacks the parameters and equations required to incorporate population dynamics and is not capable of tracking cascading losses in arthropods except at the level of taxonomic order. Therefore, it is possible that we are underestimating the risk and number of secondary extinctions, particularly since cascading effects from birds often influence arthropods (Murakami & Nakano, 2000). Natural and manipulative experiments that examine specific coextinctions from species loss would overcome these limitations, such as how Jönsson and Thor (2012) conducted a CVA predicting the effect of common ash *Fraxinus excelsior* diebacks from disease on affiliated lichen communities. However, obtaining such data is generally difficult and time‐consuming.

Finally, our web does not account for birds' abilities to fly large distances, which allows them to be part of multiple food webs in disparate locations in the same period (which is referred to as a *metacommunity*) (Leibold et al., 2004). Such behavior, as observed in previous studies (Maron et al., 2006), can result in significant fluxes of nutrients that have the potential to alter ecosystems. Consequently, the loss of birds from our food web may induce secondary extinction cascades within the desert community via non‐consumptive effects (defined as the impact of animals on the growth, behavior, or development of other species, e.g., Peckarsky et al., 2008). These effects cannot be accounted for in a network based solely on predator–prey interactions (Wooten, 2020). Indeed, although researchers have known for years that non‐consumptive effects impact population dynamics in food webs (Lima & Dill, 1990; Peckarsky et al., 2008) and have conducted manipulative experiments on how they impact smaller webs (Schmitz, 2008), such research has only recently been introduced into analyses of full ecological networks (e.g., Ho et al., 2019).

## AUTHOR CONTRIBUTIONS

**Adam J. Eichenwald:** Conceptualization (equal); data curation (lead); formal analysis (lead); investigation (lead); methodology (equal); project administration (lead); validation (lead); visualization (lead); writing – original draft (lead); writing – review and editing (supporting). **Nina H. Fefferman:** Conceptualization (equal); methodology (equal); project administration (equal); supervision (equal); writing – review and editing (equal). **J. Michael Reed:** Conceptualization (equal); formal analysis (equal); funding acquisition (equal); investigation (equal); methodology (equal); project administration (equal); supervision (equal); writing – review and editing (equal).

## FUNDING INFORMATION

This research was funded by the Tufts Biological Resilience Integration Institute (NSF NRT #2021362), and the Tufts D3M program (NSF NRT #2021874).

## CONFLICT OF INTEREST STATEMENT

All authors declare that they have no conflicts of interest.

## Supporting information


Table S1

Table S2

## Data Availability

The data that support the findings of this study are available in Dryad at https://datadryad.org/stash/share/IQcQTAqOXzTyJXiQdN_jBPq6xZxYmaiUBZofKW1vFFQ. The data and source code that supports the findings of this study are also available at https://github.com/aeiche01/MojaveFoodWeb/tree/main.
